# Autism & Gluten: The Proof By Regression!

**DOI:** 10.15844/pedneurbriefs-32-9

**Published:** 2018-09-14

**Authors:** Hakim Rahmoune, Nada Boutrid

**Affiliations:** 1 Pediatrics Department, Setif University Hospital, Setif-1 University, Algeria; 2 Genetic & Nutritional Diseases Laboratory, Setif-1 University, Algeria

**Keywords:** Child, Humans, HLA-DRB1 Chains, Diet, Gluten-Free, Celiac Disease, Autistic Disorder

## Abstract

Investigators from different clinical and research centers from Paris, France studied the polymorphisms of the HLA class II loci in an autistic population. Through a case control design, they looked for the distribution of HLA class II alleles, genotypes and haplotypes in Autism Spectrum Disorders (ASD) patients meeting DSM-IV TR criteria versus healthy controls (HC).

Investigators from different clinical and research centers from Paris, France studied the polymorphisms of the HLA class II loci in an autistic population. Through a case control design, they looked for the distribution of HLA class II alleles, genotypes and haplotypes in Autism Spectrum Disorders (ASD) patients meeting DSM-IV TR criteria versus healthy controls (HC). Subsequent comparisons of the HLA genotypes from these ASD patients and healthy controls (474 and 350 respectively) were conducted. A particluar HLA haplotype (HLA-DRB1 *11-DQB1*07), stongly associated to celiac disease was more prevalent in ASD patients, versus HC (p = 0.001). Another haplotype (HLA-DRB1 *17-DQB1*02 ) was higher in HC and thus considered as protective. The authors concluded that these finding would support previous works linking gluten to autism and could open a new window of opportunity to a better understanding of ASD. [1]

COMMENTARY. This innovative, large cohort study adds a new piece to the puzzle of pathophysiology and potential therapy of ASD. Starting from the pivotal role of the HLA system in innate and adaptive immune responses and the frequent immune dysfunctions in ASD, the researchers analyzed the distribution of the HLA-class II genes in a sample of ASD patients versus healthy controls. Interestingly, they found the HLA-DRB1*11-DQB1*07 haplotype in 14.53% of the ASD population versus 8.7% in healthy controls. This difference was statistically significant with a *p* value = 0,00172 and an Odds Ratio= 1,75 (Confidence Interval =1,24–2,47).

This peculiar haplotype is the most important genetic background predisposing to celiac disease (CD), and is even included in the algorithm of the last European Society for Pediatric Gastroenterology, Hepatology, and Nutrition guidelines for the diagnosis of CD [2]. In fact, CD patients present many gastro-intestinal disorders similar to ASD patients, and unbalanced gastrointestinal microbiota (i.e. dysbiosis) and increased intestinal permeability are widey described in children with ASD [3]. In addition, ASD patients exhibit an elevated auto-antibody response to tissue transglutaminase-2, the key enzyme of the inflammatory cascade in CD [4] and gluten free diet is frequently cited in the treatment pannel of autistic disorders [5].

We think that such results pave the way to a new era of genotype-phenotype correlations: at least a subtype of autism would be considered as a gluten-related disorder and patients could be offered an adapted diet via an evidence-based medicine.

## Disclosures

The author(s) have declared that no competing interests exist.
